# Polymeric Nanowires for Diagnostic Applications

**DOI:** 10.3390/mi10040225

**Published:** 2019-03-29

**Authors:** Hendrik Hubbe, Eduardo Mendes, Pouyan E. Boukany

**Affiliations:** Department of Chemical Engineering, Delft University of Technology, Van der Maasweg 9, 2629HZ Delft, The Netherlands; h.m.k.hubbe@tudelft.nl (H.H.); e.mendes@tudelft.nl (E.M.)

**Keywords:** Affordable biosensor, polymeric nanowire, bio-microfluidics, biosensor, bioelectronics, bio-diagnostics

## Abstract

Polymer nanowire-related research has shown considerable progress over the last decade. The wide variety of materials and the multitude of well-established chemical modifications have made polymer nanowires interesting as a functional part of a diagnostic biosensing device. This review provides an overview of relevant publications addressing the needs for a nanowire-based sensor for biomolecules. Working our way towards the detection methods itself, we review different nanowire fabrication methods and materials. Especially for an electrical signal read-out, the nanowire should persist in a single-wire configuration with well-defined positioning. Thus, the possibility of the alignment of nanowires is discussed. While some fabrication methods immanently yield an aligned single wire, other methods result in disordered structures and have to be manipulated into the desired configuration.

## 1. Introduction

One-dimensional nanostructured materials, namely nanowires, have a strong potential to provide a valuable platform for sensing of biomolecules and pathogens when integrated in affordable devices. To date, the search for one-dimensional nanostructures of high quality materials with control of the diameter, length, composition, and phase has enabled some strong advances with their incorporation as functional parts of integrated devices [1,2,3,4,5]. While this review focuses on polymeric nanowires, the interested reader will find detailed information on silicon nanowires [1,2,4,5,6], III-nitride semiconductor nanowires [7], and carbon nanotubes [2,8,9] in the cited literature.

From all possible materials that can be used for diagnostic purposes, polymeric nanowires are promising candidates. Ease of formulation, biocompatibility, their suitability for decoration with biomolecules, and the availability of electrically-conductive materials are advantages that can be utilized due to the highly precise chemistry of synthetic polymers, which is very well established by now [10,11]. Conductive properties of polymers, however, differ strongly from other typical materials used in system integration electronics, such as metals and semiconductors. Examining the literature of nanowire system integration, it appears that the a priori, lower conductive properties of polymers in relation to typical materials used in integrated electronics has left polymer nanowires under-investigated for such purposes. Finding new solutions and approaches, resistance (or impedance) measurements using conductive polymers have also to be considered, besides the more common optical read-out approaches.

The goal of this review is to provide the state-of-the-art status of polymeric nanowires for diagnostic applications in integrated electronics. The steps associated with the production of a polymeric nanowire-integrated device are: synthesis or formulation of the nanowire; deposition of the nanowire on the device, a processing step, which is also associated with the ability to align such a nanowire in a well-defined direction of the device; finally, a detection method with enough sensitivity for the purpose of the diagnostic application. It happens that some nanowire synthesis methods intrinsically impose an orientation on the deposited nanowires, while others allow for the use of deposition and alignment of the nanowire as a separate step. In view of this, this article is divided into several sections. We start with “Randomly-Aligned Nanowires”, indicating that the method of producing the nanowire results in a random orientation of the wires. A second section on “Aligned Nanowires” describes the methods of integration, where the synthesis and deposition of nanowires are achieved during synthesis or using two separate steps. Finally, various methods used in the detection of biomolecules and bacteria based on polymeric nanowires of the preceding sections are examined, together with their sensitivity. A short conclusion on the most promising strategies and their limitations is presented at the very end.

## 2. Randomly-Aligned Nanowires

Random polymers are composed of long molecules, and the systems are flexible and elastic. If not confined in any way, the nature of the material will lead to disorder of the nanowire structure [12,13]. This section presents nanowires of interest for a diagnostic application, which are randomly oriented after synthesis. Strategies for alignment are discussed in a later section.

### 2.1. Electrospinning

Electrospinning is an old technique, long established in the textile industry and with recent advances into the biomedical field. Interest in electrospinning arises from the huge variety of materials that can be spun into fibers, with diameters in the nanoscale well possible. Intensive reviewing of the various possibilities including hierarchically-structured fibers, but exceeding our review’s scope have been discussed in the review of Yang et al. [14].

Researchers made composite materials from this simple method, producing conductive nanofibers. Recently, Lee et al. [15] presented a highly-conductive electrospun polyethylene oxide (PEO) nanowire carbon nanotube (CNT) composite, using poly(styrene sulfonic acid graft aniline) (PSS-g-ANI) as an amphiphilic surfactant to create a stable solution of the CNT and the polymer. High electrical conductivity (1570 S/m) was reported, comparing well to the intrinsic conductivity of conductive polymers varying from 10^−14^–10^2^ S/cm [16].

**Figure 1:**
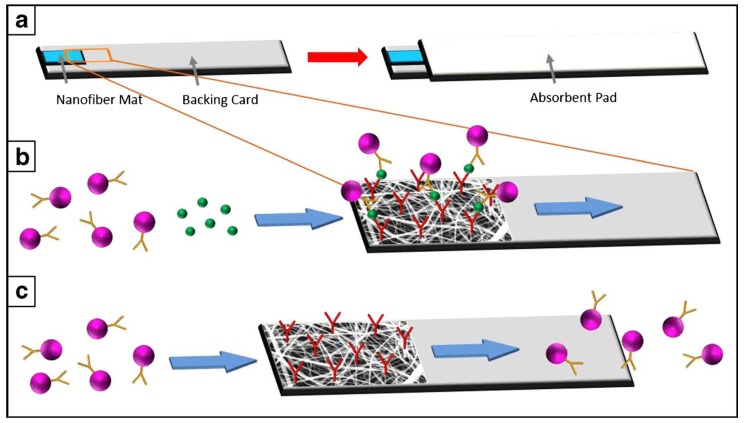
Illustration of a lateral flow assay using an electrospun nanofiber mat instead of the classic nitrocellulose membrane: (**a**) sample flows through the mat into the absorbent pad; (**b**) *E. coli* (green) are captured by the antibodies (red) on the mat, and following horseradish peroxidase (HRP), linked antibodies (pink/orange) are immobilized, catalyzing a colorimetric signal in place when the HRP substrate is added; (**c**) if *E. coli* is not present in the sample, no binding of the secondary antibody can occur, and the enzyme is transferred to the absorbent pad. Reprinted by permission from Springer Nature: Springer *Anal. Bioanal. Chem.* [17]. Copyright 2013.

Reinholt et al. [17] showed that an electrospun polylactic acid-polyethylene glycol (PLA-PEG) mat without further advancements is comparable in use as a lateral flow assay to a classic nitrocellulose membrane (Figure 1). They demonstrated its use as a matrix in an antibody-based *Escherichia coli* immunoassay, achieving a limit of detection of 3.8 × 10^6^ CFU/mL, which is comparable to those seen in the literature for nitrocellulose membranes [18,19].

**Figure 2:**
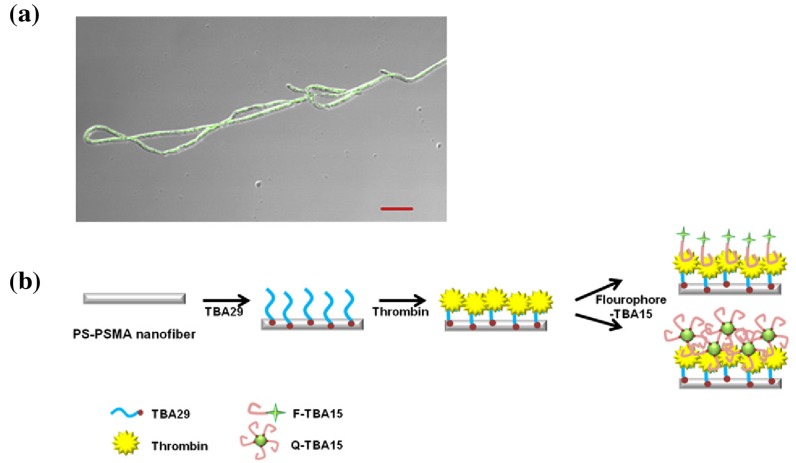
(**a**) Composite microscope picture combining the brightfield and fluorescence of anti-thrombin aptamer sandwich on nanofiber with a quantum dot-labeled secondary aptamer (scale bar: 20 μm); (**b**) schematic of the nanowire-based anti-thrombin sandwich assay (TBA: thrombin-binding aptamer). Reprinted from [20], Copyright 2012, with permission from Elsevier.

Aiming at hormone/protein detection, Lee et al. [20] could demonstrate the feasibility of outfitting electrospun polystyrene-poly(styrene-co-maleic anhydride) (PS-PSMA) with aptamers (Figure 2). This is of special interest, as the maleic acid contained in the spun wire comprises a bioconjugation compatible linker. Although more of a microfiber in diameter (compare Figure 2), reduced sizes might well be achieved in the future or not even necessary for the envisaged diagnostic application. Lee et al. had it react with streptavidin in a simple buffer solution overnight and attached the primary biotin-linked aptamer in the next step. In the presented aptamer sandwich immunoassay, the group was able to achieve thrombin detection down to a concentration of 10 pM, using secondary aptamers conjugated to quantum dots.

The process of electrospinning does not yield oriented wires. New setups tried to overcome this disadvantage, such as aligning the wire to a magnetic field while still flying [21] or reducing the whipping motion by adjusting the electrical field to a lower strength and higher uniformity and mechanically pulling the wire towards the collector [22]. Although a linear pattern of wires can be created, this process is not able to define the position of each single wire precisely. Without any further processing, the result is still that of randomly-placed wires with a common orientation and mean density.

**Figure 3:**
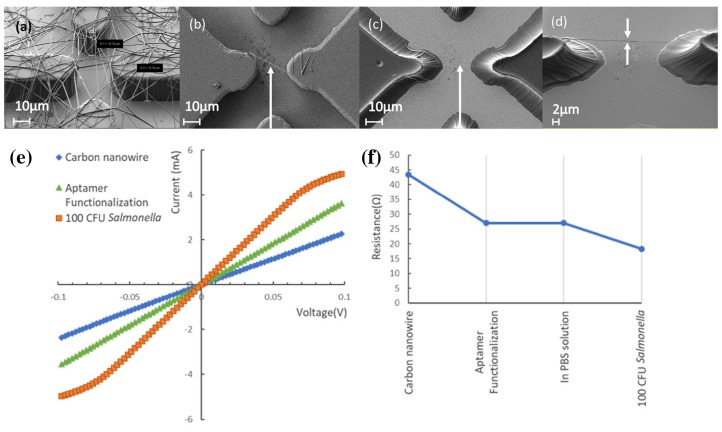
(**a**) SU8 photoresist electrode structure with electrodeposited SU8 photoresist nanowires, (**b**) after UV curing and development, and (**c**,**d**) finally carbonized in a furnace; (**e**,**f**) resistance changes measured in the nanowire after decoration with the aptamer and addition of *Salmonella* bacteria. Reprinted from [23], Copyright 2018, with permission from Elsevier.

A unique strategy for creating aligned wires was shown by Thiha et al. [23] (Figure 3). SU8 photoresist was electrospun onto an electrode structure, creating an area of randomly-distributed wires as typical for electrospinning. They illuminated the small gap in between the electrodes with UV light, so that only a part of a wire spanning this gap was cured. Any neighboring wires were dissolved in the following development step. The authors reported that in five repetitions with sets of 24 electrode structures each, 80% showed 1–3 suspended wires, 20% of these resulting in a single-wire configuration. Perhaps this rate can be improved, if the electrospinning is combined with an alignment technique such as the aforementioned magnetic field induction [21]. As both the electrode and wire are carbonized in a furnace afterwards, this process results in an electrical contact of a conductive (then) carbon nanowire.

Overall, electrospinning has shown a high variety of materials and possibilities to combine them in composites, making it a promising process for the production of polymeric nanowires. Reliable processes for decoration of the wires have to be established. A functionalization of the respective polymer that is easily compatible with a standard bioconjugation method such as N-hydroxysuccinimidyl (NHS) esters or a bioorthogonal strategy such as Copper(I)-catalyzed Azide-Alkyne Cycloaddition (CuAAC) “click-chemistry”, which were intensively reviewed by Zheng et al. [24], is desirable. The main issue remains the alignment: extra steps are needed to align or remove the usually random fibers. A later section will discuss possible methods for alignment of such fibers.

### 2.2. Self-Assembly of Micelles

An elegant way of producing nanowires of well-defined diameter and chemical properties is self-assembly of worm-like micelles. In contrast to spherical micelles, the choice of polymer favors the assembly of molecules in the cylindrical body in lieu of a spherical end-cap, resulting in the growth of a micellar wire (further details and examples of stimuli-responsive worm-like micelles were given in [25]). Polymeric micelles are strong candidates for nanowires, as long as they can be either quenched or assembled during the dewetting process, exhibiting in both cases hundreds of microns in length [26]. So far, only quenched micelles grown out of equilibrium exhibit such dimensions. Furthermore, it has been shown that quenched micelles, containing a glassy core, exhibit such a length without branching. Zhang et al. [27] presented such micelles, with lengths of up to 250 μm. The amphiphilic nature of the building blocks opens up many options when it comes to integration of different molecules into the core while keeping the solubility in a solvent unimpeded. Another advantage is that once the monomers are available, processes for forming the wires are rather facile.

**Figure 4:**
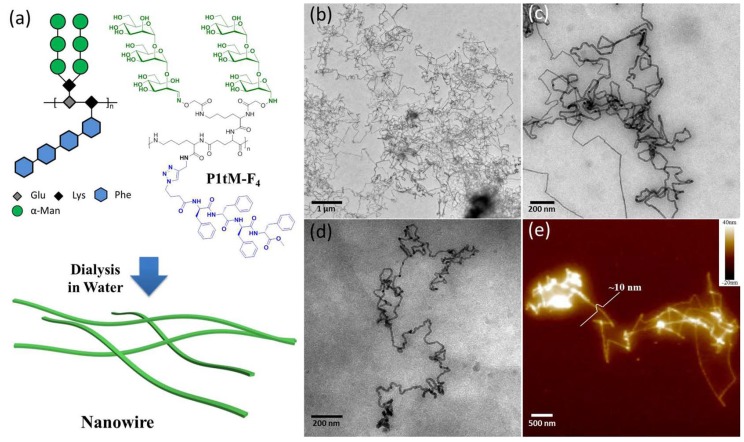
One variant of self-assembled micelle nanowires (P1tM-F_4_) created from amphiphilic glycopolypeptide brushes in water. (**a**) Chemical structure and illustration of nanowires, (**b**,**c**) TEM images in different magnifications, (**d**) cryo-TEM image and (**e**) AFM image; as no alignment is performed on the nanowires, they deposit in a random configuration of disorder. Reprinted with permission from ACS [28].

Liu et al. [28] presented a library of alternating amphiphilic glycopolypeptide brushes (AAGBs), where varying ratios of sugar and amino acid units lead to self-assembly of micelles, nanoribbons, or nanowires. Figure 4 depicts an example of a glycopolypeptide configuration resulting in nanowire self-assembly. The material seems to be a very promising candidate for good biocompatibility due to the building blocks naturally occurring in the body. Biofunctionalization would have to be investigated.

Glazer et al. [29] showed a polystyrene-polyethylene oxide block-copolymer (PS-b-PEO) micelle nanowire that self-assembles using an immiscible solvent process, as previously introduced by Zhu et al. [30]: the polymer is dissolved in chloroform, and droplets of the solution are added to water, leading to a rearrangement of the molecules to form a stable micelle nanowire in the water phase, once the chloroform evaporates. The resulting nanowires have a hydrophilic corona and hydrophobic core, showing lengths of hundreds of micrometers with a diameter of approximately 60 nm. They mention the incorporation of a hydrophobic dye (DiI) inside the micelles, showing an example for using the unique amphiphilic nature of micelle-based nanowires. Without further processing, the nanowires are disordered (see Section 3.2).

Another example of micelle functionalization was demonstrated by Nie et al. [31]. They incorporated CdS nanorods into PS-b-PEO worm-like micelles by using a surfactant (hexadecyl trimethyl ammonium bromide).

The interested reader will find further information in comprehensive reviews covering the recent advances in self-assembly of amphiphilic block and graft copolymers and their biomedical applications in [32,33,34,35,36,37].

## 3. Aligned Nanowires

Developing low-cost strategies to order, assemble, and align macromolecules (polymeric chains) is a challenging task, due to the random conformation of polymeric chains in solution. However, if the advantages of single-wire configurations are desired for sensing applications, alignment is crucial. The main advantage of the single-wire configuration is the possibility of electrical contacting and better defined signal density. Alignment in this sense can mean two things: the change of orientation of a given wire, but also the direct creation of a wire structure in the preferred orientation and place. Many techniques have been thoroughly reviewed by Su et al. [38,39]. The following sections focus on vertical nanowires, micropillar dewetting, and electrosynthesizing.

### 3.1. Vertical Nanowires

Vertical nanowires represent an ideal variant of aligned nanowires. A major advantage for diagnostic applications can be the very high surface area achievable with the dense packing possible in such geometry.

A top-down approach to achieve vertical polymer wires was demonstrated by Fang et al. [40]. They created vertical nanowire arrays in UV-transparent and UV-absorbent polymers through laser interference patterning (LIP) and inductive coupled plasma (ICP) etching. Diameters increase with the pattern periodicity and vary from 100 nm with a 500 nm period up to 500 nm for the 2.5 μm period array. Aspect ratio and height are not explicitly discussed, although the authors claimed that elongated ICP etching would not change the LIP pattern and so increase the aspect ratio. The group previously presented ICP-based etching of nanowires into diverse organic materials, achieving wire lengths of several micrometers [41,42].

A vertical architecture can be challenging if it comes to electrical contacting, as connecting the top end of a free-standing wire is not a trivial procedure in these dimensions. Vlad et al. [43] showed the vertical growth of PANI nanowires, using a PMMA template (see Figure 1 in [43]). Using this template, they could also facilitate electrical contact on top of the wires. The nanowire height was 500 nm for cross bar latched arrays with top and bottom metal electrode contacts.

Polypyrrole (PPy) nanowires appear suitable for work with cells, due to their property of swelling on application of electrical fields, potentially allowing controlled release of substances incorporated into the wire, but also the triggered detachment of cells adhered to it [44]. This has been used by the same group in Hong et al. [45] to demonstrate circulating tumor cell (CTC) capture and release using vertical PPy nanowire arrays (see Section 4.2). As a sample usually contains only low concentrations of the targeted cells, capturing them on a small surface area enhances the signal of a subsequent detection, allowing here a very low detection limit of 10 cells/mL. To facilitate binding of the capture antibody, disulfide-biotin was incorporated into the PPy nanowires during electrochemical deposition of the pyrrole. It allowed for ligation of the biotinylated capture antibody through streptavidin addition and offered a second option for detaching captured cells by cleaving the sulfide bond using glutathione instead of applying a voltage.

### 3.2. Micropillar Dewetting

**Figure 5:**
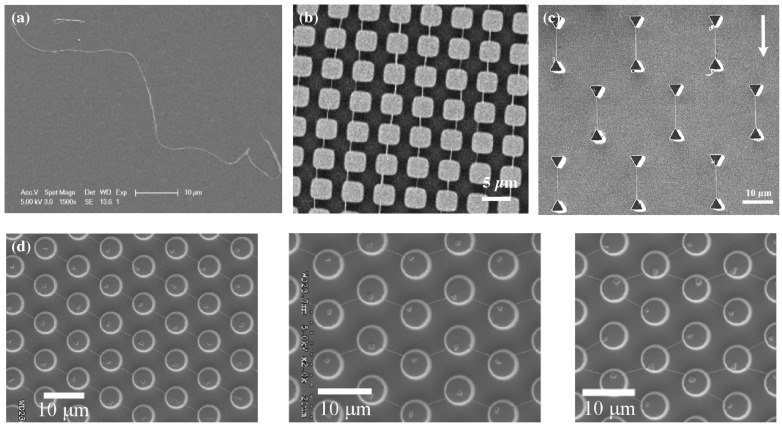
(**a**) Disordered deposition of a micellar nanowire on an unstructured surface, compared to (**b**) aligned nanowires after dewetting of microstructures (square pillars of 3.5 μm in width, 2 μm in gap, and 3 μm in height) [29]. (**c**) Variation of the microstructure geometry yields different nanowire formations [48], also seen in (**d**), where a different flow direction angle of receding fluid in each picture additionally influenced the layout of resulting nanowires [47]. (**a**–**c**) reprinted from [29,48] with permission from Wiley; (**d**) reprinted from [47] with permission of AIP publishing.

Wetting is a common phenomenon that can often be found in our everyday life activity from swimming on a beach to effects in ion transport. The dewetting process of liquids, containing macromolecules on micro-patterned substrates, has become an effective strategy to form large arrays of polymeric nanowires. Various types of polymeric nanowires including DNA, polyvinyl formal (PVF), and PS-b-PEO worm-like micelles have been successfully formed. The techniques yield large arrays of oriented wires out of polymer solutions by retracting the solution drop from micro-patterned surfaces. This has been shown in different ways (see Figure 5): Guan et al. and Lin et al. [46,47] used a solution of DNA wires, enclosing the droplet between the structured stamp and a plain surface and carefully separating the planes. Glazer et al. [29] created worm-like micelles with a PS-b-PEO block copolymer and dewetted the structured stamp removing the liquid with the tip of a tissue. Su et al. [48] created the dewetting effect by moving a droplet of dissolved polyvinyl formal over the structure with a pipette, forcing the dissolved polymer to entangle and form wires, while the solvent retracted. In a different publication, Su et al. [49] employed the same strategy for calcein, but using gravity by tilting the substrate.

Many different dewetting structures have been shown to yield various patterns of aligned wires [50]. In general, this method seems applicable to alignment of any randomly-produced nanowires available in solution. Furthermore, it has been shown that this method can be employed to hold in restraint *S. aureus* bacterial colonies and shape the architecture of their growth for biological investigations [51]. Furthermore, electrical contacting should be feasible, but has yet to be shown.

### 3.3. Electrosynthesizing

**Figure 6:**
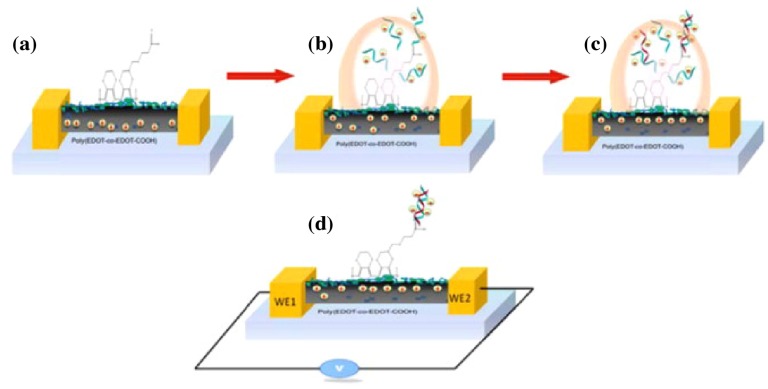
(**a**) Schematic of electrosynthesized nanowire with -COOH end groups for conjugation (**b**) with oligonucleotides (ODN) through EDC/NHS chemistry, (**c**) hybridization of target ODN, and (**d**) resistance measurement after drying of the wire. Reprinted from [52], Copyright 2012, with permission from Elsevier.

**Figure 7:**
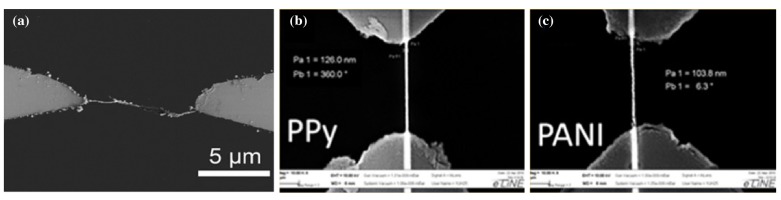
Comparison of resulting electrosynthesized wires: (**a**) PEDOT nanowire grown in between two gold electrodes [52] and (**b**,**c**) PPy and PANI nanowires grown in a confining PMMA nanochannel [53]. Reprinted from [53], Copyright 2013 and [52], Copyright 2012, with permission from Elsevier.

The process of electrosynthesizing is of interest, as it combines synthesis, alignment, and electrical contacting in one step. Different materials and processes have been presented in the past. Kannan et al. [52] showed the electrosynthesizing of a single poly(3,4-ethylenedioxythiophene) (PEDOT) wire in between two gold electrodes. They decorated it with DNA oligonucleotides specific to ovarian and breast cancer cells (Figure 6). A mixture of the monomer EDOT/EDOT-COOH, LiClO_4_, and polystyrene sulfonate (PSS) in acetonitrile was placed on the electrode gap and exposed to a square wave electric field (±7 V at 25 kHz). The resulting wires were not exactly straight and showed a high roughness, but spanned a distance of 11 μm (Figure 7).

To overcome the random fluctuations in electrogrown wires, Hu et al. [53] confined the wire growth inside a PMMA nanochannel (height 100 nm, channel diameter 100 nm) flanking the direct connection between the two electrodes (Figure 7). They showed a combined sensor chip, using electrically-conductive polymer, ZnO, and palladium wires for gas sensing (hydrogen, methanol, carbon monoxide, and nitrogen dioxide). Both a PPy and a polyaniline (PANI) nanowire were grown through electrochemical deposition in between two Ti/Au electrodes. Owing to the channel confinement, the shown wires spanning the 5 μm distance were straight. Measured widths of the wires were 126 nm for PPy and 104 nm for PANI.

**Figure 8:**
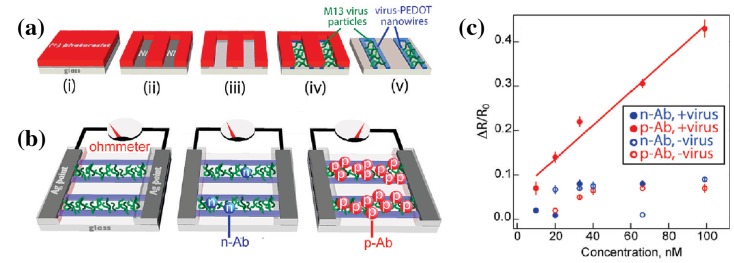
(**a**) Illustration of the synthesis of virus-containing PEDOT nanowires through lithographically-patterned nanowire electrodeposition (LPNE). (**b**) Schematic of the resulting nanowires in the final configuration; relatively low ohmic resistance when bare, also after addition of non-binding control antibodies (n-Ab), but significant rise of resistance after applying virus-binding antibodies (p-Ab). (**c**) Resistance changes for combinations of virus containing/free and binding/non-binding antibodies to demonstrate the selectivity. Reprinted with permission from [54]. Copyright 2010 American Chemical Society.

A very uncommon, yet intriguing composite of an electrodeposited polymer with integrated bacteriophages has been shown by Arter et al. [54]. Nickel electrodes were used to create thin initial layers of PEDOT through electrodeposition, facilitating the following co-deposition of PEDOT and M13 bacteriophages (see Figure 8). The nickel was removed and the resulting nanowires contacted with silver paste. The authors demonstrated the functionality of the bacteriophage surface by attaching fluorescing anti-M13 antibodies. However, most importantly, they showed significant resistance changes of the wire arrays on administration of the binding antibody, as compared to the negative control (Figure 8c). The future idea is that viruses can be modified to carry specifically-engineered binding sites on the outside, thus allowing the buildup of different diagnostic assays. This could allow label-free detection of analytes using libraries of virus variants.

## 4. Detection Strategies and Opportunities

With all previously-mentioned nanowire materials and alignment methods, the key point towards a diagnostic device is the detection strategy employed to address healthcare-relevant device needs. This last section focuses on strategies for use of polymer nanowires in detection applications. Findings are summarized in Table 1. Some considerations concerning packaging are given in the last section.

### 4.1. Optical

Polymeric fibers can be decorated with biofunctional molecules such as antibodies, aptamers, or oligonucleotides. In the most basic approach, a detection is then possible using a label such as a fluorophore or an enzyme, catalyzing a color-change reaction.

An approach using enzyme has been shown by Reinholt et al. [17], as previously mentioned. They created a lateral flow assay using an electrospun mat of PLA-PEG wires to perform a sandwich immunoassay detecting *E. coli*. The labeling secondary antibody was linked to horseradish peroxidase, a commonly-used enzyme in immunoassays, catalyzing a color-change with an added substrate. The wicking and washing time of the assay were not stated, the duration of the final color reaction was 10 min.

A fluorophore-based setup was demonstrated by Lee et al. [20]. Instead of antibodies, aptamers were used to detect the protein thrombin. Especially interesting in this setup is that the decoration of the PS-PSMA nanowire occurred using the intrinsic reactivity of the maleic anhydride part of the wire towards streptavidin. Decoration with biotin-linked aptamers can follow thereafter. The immobilization of the streptavidin on the wire surface took place in aqueous buffer solution. The approach should easily allow for any other biotin-tagged biomolecule to be used in this assay. Incubation time and washing steps amounted to ~1.5 h.

Aptamers are similar to antibodies, in that they bind specifically to the target molecule with a high specificity. They are made of sequences of either RNA or DNA. Advantages compared to protein-based antibodies are numerous. Target specificity and affinity can be selected in vitro, allowing a broader variety of targets: culturing of antitoxin antibodies for example is difficult, as it involves application of the toxin to the animal or animal cells producing the antibody. In some cases, the DNA aptamer is more stable than the antibody protein. Synthesis happens chemically with little to no batch variations and a higher shelf life. Finally, the aptamer often changes conformation when binding, an effect that can be detected with an integrated sensor. This was discussed by Lee et al. and Song et al. [55,56], where further information can also be found.

Garcia-Cruz et al. [57] presented a nanocontact printed PPy nanowire, decorated with antibodies to detect the protein interleukin-10. They could show the specific binding to the nanowire using secondary fluorophore-linked antibodies. The optical-based proof-of-principle testing took 2 h. As PPy is a conductive polymer, it will be interesting to see future applications of electrical read-outs using this setup.

### 4.2. Electrical

**Figure 9:**
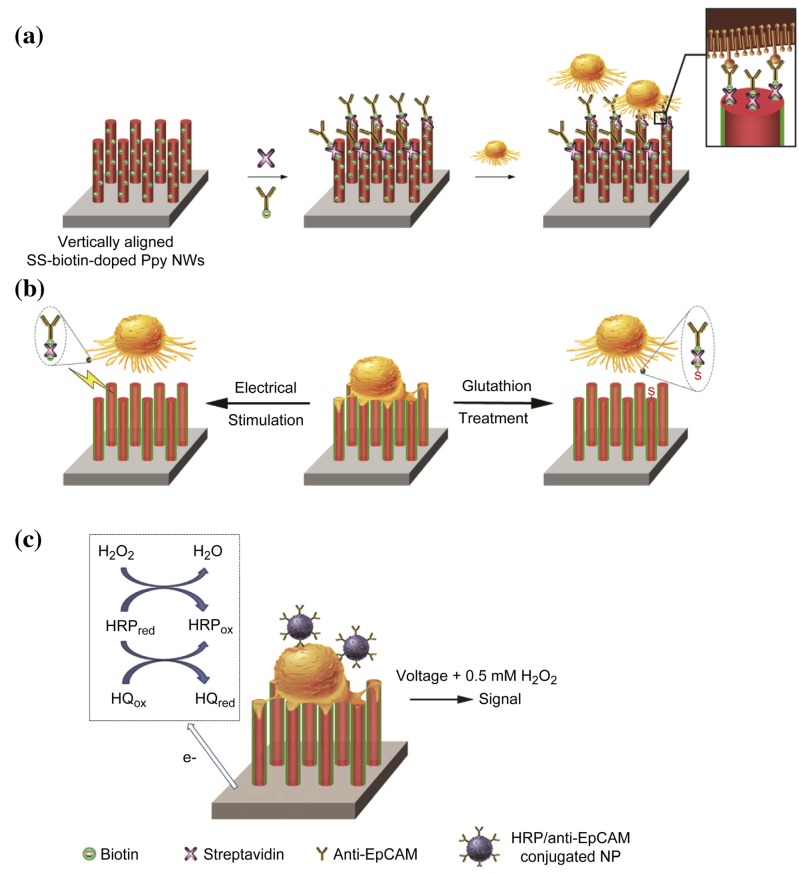
(**a**) Composition of the cell capture array: disulfide-biotin has been doped into the vertical PPy nanowires during electrochemical polymerization and linked to the biotin-linked anti-EpCAM antibodies through addition of streptavidin. (**b**) Captured cells can be released either by applying a voltage swelling the PPy or the addition of glutathione for cleavage of the disulfide bond. (**c**) Electrochemical detection after labeling of captured cells with HRP-nanoparticle-linked antibodies. Reprinted from [45], Copyright 2014, with permission from Elsevier.

The already introduced work of Hong et al. [45] used an array of vertical PPy nanowires to capture CTCs using anti-EpCAM antibodies (see Figure 9). They concluded a nine-fold higher capture of cells as compared to a flat PPy surface and were able to demonstrate a limit of detection down to 10 cells/mL using antibody-linked HRP, performing amperometric measurements. Although based on silicon nanowires, we also recommend for further reading the work of Wang et al. [59] and Lu et al. [60]. The application of microfluidics to this field has been reviewed by Chen et al. [61].

Additionally interesting in this regard is the use of vertical nanowires for sample preparation such as cell lysis, which would have to precede a detection, if the target is contained inside the cell rather than on the cell surface. An example was shown by Kim et al. [62], using vertical ZnO nanowires to lyse cells for release of contained proteins and nucleic acids. Further background on the challenges of microfluidic cell lysis was provided by Nan et al. [63].

In some cases, changes in a nanowire’s electrical resistance are measurable if the composition of the matter in the close proximity of a nanowire changes. This enables the direct detection of target molecules, as they bind to a functionalized decoration of the conductive nanowire. An additional molecular label as discussed for the optical detection methods or the electrochemical detection is not necessary.

In the previously introduced work of Kannan et al. [52], a PEDOT nanowire was decorated with oligonucleotides, which hybridize with their targeted counterparts, if contained in the sample. They proposed that the close proximity of the negatively-charged backbone of the hybridized oligonucleotide leads to an increase in immobile, surface-trapped electron holes. This results in a decrease of the wires’ conductivity. In the presented work, the wire had to be dried after application of the sample droplet and 1 h of incubation time at 42 ${}^{\circ }C$, before an electrical measurement was started.

The drying process was not necessary in the setup of Thiha et al. [23] and was performed with the wire submerged in the buffered sample solution after 5 min of incubation time. The nanowire and electrodes were integrated into a microfluidic channel. In their case, the polymer nanowire was transformed into a pure carbon wire using a furnace and high temperatures. Decorated with an aptamer, *Salmonella* bacteria were captured on the wire, and a decrease in electrical resistance can be measured directly while the wire is covered with buffer solution. The Gram-negative nature of the bacteria accounts for a high concentration of lipopolysaccharides in its membrane, creating a negative membrane charge. Bound in close distance to the wire on the aptamer, the charges induce electron holes in the carbon wire, lowering the resistance.

Zhu et al. demonstrated a different method of electrical read-out using electrochemical impedance spectroscopy, but using a flat, nanoporous electrode of a conductive polymer [58]. For this, a potential was applied to the electrochemical system, and the frequency dependence of the impedance was analyzed. This frequency dependence is especially sensitive to surface interactions. In this setup, the folding of the immobilized aptamer when binding to the target protein (estradiol) leads to a charge redistribution. The range of detection was as low as 1 fM, and the incubation time was 20 min. It will be interesting to see if this measurement method can be adapted to conductive polymer nanowire systems.

A common problem in sensing biomolecules is unspecific binding, leading to false positive signals and a higher limit of detection. Two effective approaches to optimize this aspect were developed. In the work previously discussed, Reinholt et al. [17] presented a second PLA mat, which included additions of antifouling polymer in the electrospin solution. It reduced nonspecific binding beyond detection limits, showing a possible advantage of using electrospun wires over nitrocellulose. A second example of interest was presented by Chang et al. [64]. They used In_2_O_3_ nanowires, decorated with antibodies against a tumor marker, and passivated the nanowire against unspecific binding by attaching Tween-20 molecules to the nanowire. It is an amphiphatic molecule commonly used in classical immunoassays for this purpose and should be applicable to polymeric wires as well.

### 4.3. Packaging

**Figure 10:**
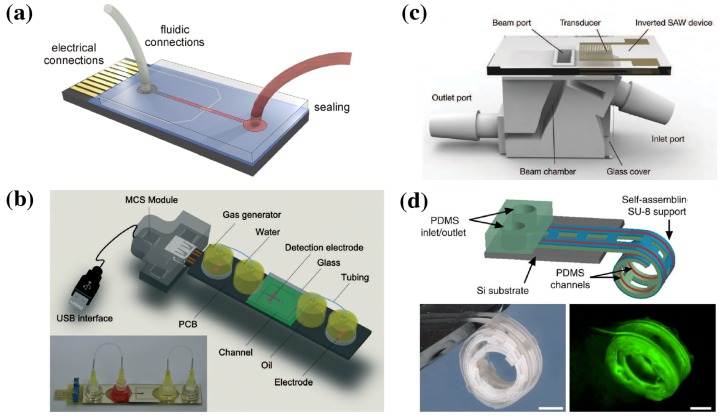
(**a**) Schematic of a microfluidic chip with sealed fluidic channels and microfluidic and electrical connections [66]. (**b**) Complex integrated microfluidic device, including reservoirs, pumps, and USB interface electronics [67]. (**c**) 3D-printed fluidic accessory [70]. (**d**) Self-assembling microfluidic channels show one of many possibilities to rethink packaging in new directions [72]. (**a**) reprinted from [66], Copyright 2015, with permission from Elsevier; (**b**,**c**) republished with permission of Royal Society of Chemistry, from [67,70], Copyright 2014; permission conveyed through Copyright Clearance Center, Inc. (**d**) Reprinted by permission from Springer Nature: Springer *Nature Communications* [72]. Copyright 2011.

Even the most advanced detection method might never make the step out of a research lab into the application world without a proper user-friendly packaging. Depending on the sensor type, it has to be tightly sealed, and electrical, fluidical, and optical connections have to be made to connect peripheries such as reservoirs, pumps, or electronics; this still poses several challenges in manufacturing and reliability (compare Figure 10) [65,66,67,68]. While in the lab, tinkering with connections can be a viable option to allow focus on the sensor technology, but even the early stage development will gain much from reliable integration. Van den Driesche et al. [69] developed a platform suitable for the sensor development phase of a microfluidic chip. It provides reliable fluidical and electrical connections to interchangeable microfluidic chips and can be useful as a development platform, although not a consumer device. Temiz et al. [66] presented an in-depth review of sealing and fluidical/electrical interconnection of microfluidic devices in general. They concluded that manifold techniques are available, but still, there is a persistent gap between the preferences of the academic researcher and mass production compatibility considerations of the industry. The most prominent example of this is the widespread use of PDMS in research labs as opposed to injection-moldable thermoplastics preferred in industry. For apparent reasons, decisions about materials used in research are often made on the basis of availability of the material and machinery, but also practical demands of the ongoing research work, such as the demand to be able to view the processes optically with a microscope. These approaches often result in a carefully optimized technology, which is not compatible with the scale-up demands of industrial companies. Early consideration of these limitations is helpful, if application in the field is desired. New fast-prototyping technologies and low-cost materials offer suitable starting points for research groups, avoiding the costly and not easily available clean room techniques [65,70]. For detailed critical discussion of 3D-printing techniques from the perspectives of microfluidics, read Waheed et al. [71].

## 5. Conclusions

During the last few decades, the field of nanowires witnessed numerous novel techniques and materials that have been developed from both the synthesis (chemistry) and processing (fabrication, manipulation, and alignment) points of view, providing a sustained advance, especially for polymer-based nanowires. However, fabrication of the nanowire, synthesis of the material composing it, and their manipulation (alignment) are imposing constraints on each other. Material properties, nanowire dimensions, and final alignment on a given substrate are therefore often interdependent.

In terms of polymer-based nanowire processing, electrospinning appears to offer the highest diversity of materials. Mixed materials, for instance, polymer blends or composites using carbon nano tubes, have been obtained, allowing for tuning of parameters such as electrical conductivity or affinity to biomolecules. Diameters down to hundreds of nanometers were demonstrated. An alternative approach is the self-assembly of micellar wires, because of the relative ease of production. This process spontaneously results in nanowires of a well-defined diameter, exhibiting lengths of several hundreds of micrometers. The amphiphilic structure of such nanowires allows for the incorporation of substances or nanoparticles into the core of the micelle in addition to possible chemical modification or binding of biomolecules to the corona. Both synthetic polymers and sugar/peptide-based materials have been used to create such structures.

Single nanowires are desired as opposed to a random mat, which is usually obtained by electrospinning. The single wire configuration is necessary either for precise electrical read-out or to guarantee a certain signal density when these are integrated into a device. To overcome this, electrosynthesizing produces wires in between two electrodes, following the direction of the applied field. In this case, alignment is an immanent component of the production process. A strong drawback, however, is the limited lengths of these wires that are only up to tens of micrometers. Furthermore, the growth process is prone to a certain degree of unevenness, which can be controlled using confined growth channels. Incorporation of viruses into these wires during the growth process is possible, greatly enhancing the potential of this technique for diagnostic assays, as viruses can be engineered to exhibit different surface structures, e.g., specific antigens.

Another promising method of nanowire alignment is the dewetting of solutions on microstructured surfaces. This can either happen with a polymer solution forming nanowires connecting the microstructures during the process of dewetting or using a solution of already prepared nanowires, which then align into the desired pattern on the microstructure during dewetting. Of special interest would be a combination of this mass alignment dewetting process and the numerous possible materials to be gained from electrospinning: while electrospinning lacks a reliable single-wire alignment, it offers access to very promising materials, composites, and blends. In combination, both techniques could well achieve a single nanowire configuration with possible electrical contacting.

For detection of biomolecules and microorganisms, approaches rely on the use of antibodies, aptamers (their synthetic equivalent), or DNA oligonucleotides. The strategy of incorporating specific viruses into the wire should also be mentioned in this context. The read-out of sensing setups often takes place in a two-step approach: adding a secondary antibody (label), which attaches to any bound target molecules and carries a fluorophore or an enzyme to catalyze a color reaction (known as sandwich immunoassay). Finally, it has been demonstrated that molecules or particles attached to the capture probes on the polymer-based nanowire are detectable using electrical conductivity readouts. Analyzing the frequency behavior (impedance spectroscopy), further advancements in terms of sensitivity might also be possible, but these aspects still remain to be investigated for polymeric nanowires. Further effort has to be made for evaluating the long-term stability of polymer nanowire-based sensors in the sense of shelf-life and storage.

## Figures and Tables

**Table 1:** Overview of relevant diagnostic setups and their range of detection.

Publication	Target	Sensor	Read-Out	Range of Detection
Thiha et al. [23]	*Salmonella* bacteria	Aptamer on carbon wire	Resistance change while wet	10 CFU/mL
Reinholt et al. [17]	*E. coli* bacteria	Antibody on nanowire mat	Colorimetric	3.8 × 10^6^ cells/mL
Hong et al. [45]	Circulating tumor cells	Antibody on PPy	Amperometric	10 cells/mL
Kannan et al. [52]	DNA oligo	DNA oligo on PEDOT	Resistance change after drying	0.1 fM
Garcia-Cruz et al. [57]	Interleukin-10 protein	Antibody on PPy	Fluorescence (for proof-of-concept)	250 ng/mL
Lee et al. [20]	Thrombin protein	Aptamer on PS-PSMA	Fluorescence	10 pM with QD
Zhu et al. [58]	Estradiol protein	DNA Aptamer on Py-co-PAA membrane	Electrochemical impedance spectroscopy	1 fM

## Abbreviations

The following abbreviations are used in this manuscript:

CFU	Colony-forming units
CNT	Carbon nanotube
CTC	Circulating tumor cells
EDC	1-Ethyl-3-(3-dimethylaminopropyl)-carbodiimide
HRP	Horse radish peroxidase
ICP	Inductive coupled plasma
LIP	Laser interference patterning
NHS	N-hydroxysuccinimide
PANI	Polyaniline
PEDOT	Poly(3,4-ethylenedioxythiophene)
PEG	Polyethylene glycol
PEO	Poly ethylene oxide
PLA	Polylactic acid
PMMA	Poly(methyl methacrylate)
Poly(Py-co-PAA)	Poly(pyrrole-co-pyrroleacrylic acid)
PPy	Polypyrrole
PS	Polystyrene
PSMA	Poly(styrene-co-maleic anhydride)
PSS	Poly(styrene sulfonate)
PSS-g-ANI	Poly(styrene sulfonic acid graft aniline)
